# The Lymphotoxin *β* Receptor Is Essential for Upregulation of IFN-Induced Guanylate-Binding Proteins and Survival after *Toxoplasma gondii* Infection

**DOI:** 10.1155/2017/7375818

**Published:** 2017-08-06

**Authors:** Kristina Behnke, Ursula R. Sorg, Helmut E. Gabbert, Klaus Pfeffer

**Affiliations:** ^1^Institute of Medical Microbiology and Hospital Hygiene, Heinrich-Heine-University Düsseldorf, 40225 Düsseldorf, Germany; ^2^Molecular Medicine II, Heinrich-Heine-University Düsseldorf, 40225 Düsseldorf, Germany; ^3^Institute of Pathology, Heinrich-Heine-University Düsseldorf, 40225 Düsseldorf, Germany

## Abstract

Lymphotoxin *β* receptor (LT*β*R) signaling plays an important role in efficient initiation of host responses to a variety of pathogens, encompassing viruses, bacteria, and protozoans via induction of the type I interferon response. The present study reveals that after *Toxoplasma gondii* infection, LT*β*R^−/−^ mice show a substantially reduced survival rate when compared to wild-type mice. LT*β*R^−/−^ mice exhibit an increased parasite load and a more pronounced organ pathology. Also, a delayed increase of serum IL-12p40 and a failure of the protective IFN*γ* response in LT*β*R^−/−^ mice were observed. Serum NO levels in LT*β*R^−/−^ animals rose later and were markedly decreased compared to wild-type animals. At the transcriptional level, LT*β*R^−/−^ animals exhibited a deregulated expression profile of several cytokines known to play a role in activation of innate immunity in *T. gondii* infection. Importantly, expression of the IFN*γ*-regulated murine guanylate-binding protein (mGBP) genes was virtually absent in the lungs of LT*β*R^−/−^ mice. This demonstrates clearly that the LT*β*R is essential for the induction of a type II IFN-mediated immune response against *T. gondii*. The pronounced inability to effectively upregulate host defense effector molecules such as GBPs explains the high mortality rates of LT*β*R^−/−^ animals after *T. gondii* infection.

## 1. Introduction

Core members of the tumor necrosis factor (TNF)/TNF receptor (TNFR) superfamily such as TNF and lymphotoxin (LT) *β* and their receptors TNFRp55 and LT*β*R are important mediators of innate immune responses and are considered to be essential for controlling pathogens [1–6]. It has been demonstrated that LT*α*, TNF, and TNFRp55 but not TNFRp75 are vital for host defense against the intracellular parasite *Toxoplasma gondii* [2, 7, 8]. Although the LT*β*R has been shown to play an important role in the defense against *Listeria monocytogenes* and *Mycobacterium tuberculosis* [5] as well as CMV [9], it is still unclear, however, whether signaling via the LT*β*R also contributes to an effective host response to *T. gondii. T. gondii*, a member of the phylum Apicomplexa, is an obligate intracellular parasite [7, 10]. Definitive hosts in which sexual reproduction occurs are felids. Due to low host specificity, *T. gondii* is able to infect most warm blooded mammals and prevalence in humans is estimated 30–70% throughout the world [11, 12]. In immune competent hosts, *T. gondii* infection elicits a protective immune response that may initially, in the acute phase, cause mild flu-like symptoms which then resolve [13]. As specific host immune mechanisms set in, *T. gondii* forms tissue cysts (stage conversion), in humans and mice preferably in brain and muscle tissue, and transition into the symptomless, chronic form of toxoplasmosis is effected, in which cysts persist lifelong [14]. In immune incompetent hosts, primary *T. gondii* infection may have severe and sometimes lethal consequences such as pneumonia or encephalitis [13, 15]. Furthermore, existing, chronic *T. gondii* infection may be reactivated in immunocompromised hosts such as AIDS patients or recipients of immunosuppressive drugs with similar repercussions [16, 17]. In addition, primary infection during pregnancy may, via placental transmission of the parasite, lead to fetal pathology, including irreversible neurological defects and, in the worst case, termination of pregnancy [13, 18, 19]. It has been demonstrated that innate immune responses are vital for the efficient control of *T. gondii* [20–22]. Although *T. gondii* lacks classical viral and bacterial pathogen-associated molecular patterns, unique protozoan-associated molecules such as GPI-anchors and profilin are recognized via toll like receptors (TLRs) [23–25]. TLR2 and TLR4-mediated signaling induces secretion of IL-12 and TNF by macrophages, and TLR11 or TLR12-mediated signaling induces secretion of IL-12 by CD8*α*^+^ dendritic cells (DC) [22]. IL-12 in turn induces secretion of IFN*γ* by NK cells [26, 27]. Besides being required for the induction of T cell responses, IFN*γ* mediates various innate effector mechanisms such as induction of IDO and production of reactive oxygen species and NO in *T. gondii* infection [28–31]. Another important effect of IFN*γ* is the induction of IFNy-inducible genes such as immunity-related GTPases (IRGs) and guanylate-binding proteins (GBPs) [32–34]. It has been demonstrated in mouse models that murine (m)GBPs, a family of 65 kDa guanylate-binding proteins, play an important role in host defense against intracellular pathogens such as *T. gondii* [35–37] and *Neospora caninum* [38]. mGBPs are highly induced via IFN*γ* after infection and are localized in intracellular vesicle-like structures. mGBP1, mGBP2, mGBP3, mGBP6, mGBP7, and mGBP9 relocate to the parasitophorous vacuole of *T. gondii* after entry of the pathogen into the cell [35]. The importance of mGBPs for the efficient control of *T. gondii* is underscored by findings that mice deficient for mGBP2 or showing a deletion of a cluster of mGBPs (1, 2, 3, 5, and 7) are more susceptible to *T. gondii* infection [35–37, 39]. The present study demonstrates that LT*β*R-deficient mice likewise show dramatically reduced survival after *T. gondii* infection, most probably due to an inability to induce appropriate IFN*γ* responses and a marked failure to adequately upregulate mGBPs.

## 2. Materials and Methods

### 2.1. Animals

This study was carried out in strict accordance with the German Animal Welfare Act. The protocol was approved by the North Rhine-Westphalia State Agency for Nature, Environment and Consumer Protection (Permit number 84-02.04.2011.A394). All efforts were made to minimize suffering of laboratory animals. LT*β*R^−/−^ mice were generated as described previously [40] and had been backcrossed for at least 10 generations onto a C57BL/6 background. Wild-type (WT) littermates were used as controls. Mice were housed under specified pathogen-free conditions in the animal facility of the Heinrich Heine University of Düsseldorf and were between 10 and 12 weeks of age at the time of infection. *T. gondii* strain ME49 was used for all experiments and maintained in the CD1 mouse strain purchased from Charles River Breeding Laboratories.

### *2.2.T. gondii* Infection

ME49 cysts were isolated from CD1 mice 6 weeks after infection as described previously [41]. Briefly, the murine cerebrum was homogenized by passaging through successively thinner cannulas. A first centrifugation step (5 min, 60 ×g, 22°C) removed cell debris. The pellet was then resuspended in PBS (Invitrogen, Karlsruhe, Germany), and an underlayer of Ficoll Paque™ Plus (GE Healthcare, Munich, Germany) was added before centrifugation (500 ×g, 25 min, 22°C, without brakes). The pelleted cysts were counted and resuspended in the appropriate amount of PBS. Infections were carried out by intraperitoneally injecting either 20 or 40 cysts (as indicated) of *T. gondii* ME49 in a volume of 0.2 mL PBS.

### 2.3. Blood and Tissue Processing

Mice were anaesthetized with 100 mg/kg Ketamin and 10 mg/kg Xylazine (both *Vétoquinol GmbH, Ravensburg, Germany*) and bled via the *vena cava inferior* on the days post infection (p.i.) as indicated. Serum was obtained by coagulating the blood (30 min at room temperature) and collecting the serum after two centrifugation steps (10 min, 8000 ×g). The brain, lung, liver, and spleen were removed, rinsed in PBS, and weighed. To determine cell numbers, spleens were collected, digested with collagenase D (Sigma-Aldrich, Taufkirchen, Germany) for 30 min in DMEM/10% FCS, and passed through a 40 *μ*m cell strainer (BD Biosciences, Heidelberg) before lysis of red blood cells with Erylysis buffer (Morphisto, Frankfurt am Main, Germany).

### 2.4. Histology

Formalin-fixed and paraffin-embedded tissue blocks of the isolated organs were collected; 1 *μ*m sections were cut, transferred onto glass slides, and stained with a standard hematoxylin/eosin protocol.

### 2.5. Serum Biochemistry and Cytokine Quantification

Serum was tested for concentrations of aspartate transaminase (AST), bilirubin, and lactate dehydrogenase (LDH) using the automated biochemical analyzer Spotchem EZ SP-4430 (Arkray, Amstelveen, Netherlands) and the Spotchem EZ Reagent Strip Liver-1 (Arkray). Commercially available ELISA kits were used to quantify serum TNF, IL-4, IFN*γ* (R&D Systems, Minneapolis, MN), and IL-12p40 (BioSciences, Heidelberg, Germany) levels. NO concentrations were analyzed using the Total Nitric Oxide and Nitrate/Nitrite Kit from R&D Systems.

### 2.6. Quantitative RT-PCR

Total RNA from single cell suspensions of lung tissue was isolated using TRIzol reagent (Invitrogen Life Technologies) according to the manufacturer's protocol. First-strand cDNA synthesis was performed using 3 *μ*g of total RNA with Moloney murine leukemia virus reverse transcriptase and oligo (dT) primer (both Invitrogen Life Technologies). RT-PCR (40 cycles) was performed in triplicate. Primer and probe sequences (listed in Table 1) were synthesized by Metabion (Martinsried, Germany) and based on the conventional TaqMan Probe finder software (TIB MOLBIOL, Berlin, Germany) for mGBP6, mGBP8, and mGBP9 and the Universal ProbeLibrary (Roche, Mannheim, Germany) for all other genes. The PCR primer sets used spanned at least one intron to avoid detection of genomic DNA. Results are expressed relative to expression in uninfected WT mice and normalized to *β*-actin (2^−ΔΔCT^).

### 2.7. Statistical Analysis

Quantifiable data are expressed as means ± SD. Statistical analysis was performed using the GraphPad Prism 5.01 software for Student's *t*-test.

## 3. Results

### 3.1. LT*β*R^−/−^ Mice Show Increased Susceptibility to Infection with *T. gondii* (ME49)

**Figure 1:**
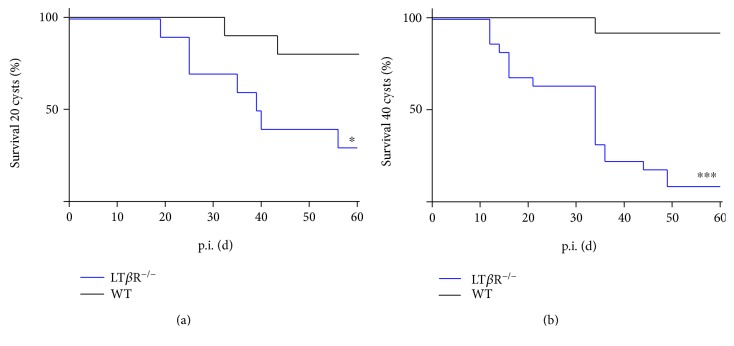
LT*β*R^−/−^ animals show significantly reduced survival after infection with *T. gondii* (ME 49) cysts compared to WT animals. WT and LT*β*R^−/−^ animals were infected i.p. with (a) 20 cysts (WT: *n* = 10, LT*β*R^−/−^: *n* = 10) or (b) 40 cysts (WT: *n* = 12, LT*β*R^−/−^: *n* = 22) of *T. gondii* (ME49) freshly isolated from the brains of CD1 mice. ^∗^*p* < 0.05, ^∗∗∗^*p* < 0.001.

It has been demonstrated that the LT*β*R plays a role in controlling infections with intracellular pathogens such as *M. tuberculosis* and *L. monocytogenes* [5]. To determine whether the LT*β*R is also required to contain infections with *T. gondii*, LT*β*R^−/−^ mice were infected with 20 or 40 cysts of the ME49 strain of *T. gondii* (Figure 1). Initially, mice were challenged with 20 cysts (*i.p.*) and significantly decreased survival could be observed (Figure 1(a)). Interestingly, LT*β*R^−/−^ mice survived the acute phase of infection and only started succumbing to the infection in the early chronic phase on day 19 with an overall survival of 30%. WT littermates started dying considerably later (day 32) and showed an overall survival rate of 80%. After infection with 40 cysts of *T. gondii* ME49, LT*β*R^−/−^ mice started to succumb to infection by day 12 and overall survival was 9.1%. In contrast, WT mice did not show earlier onset of death (day 34) and an overall survival rate of 90% (Figure 1(b)). These data clearly indicate that the LT*β*R plays a major role in surviving *T. gondii* infections.

### 3.2. LT*β*R^−/−^ Mice Show Marked Exacerbation of Organ Pathology

**Figure 2:**
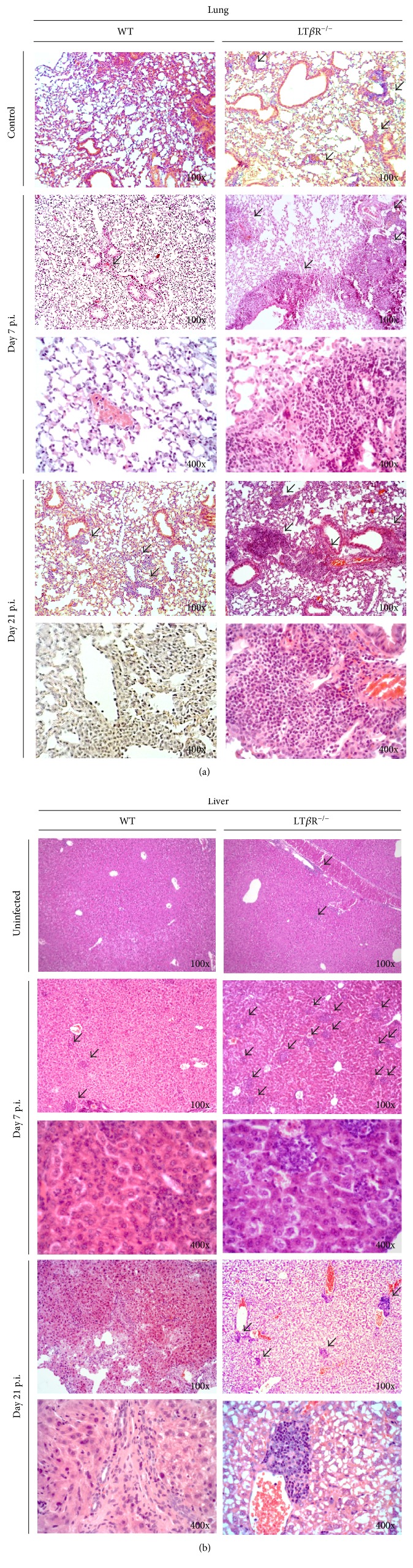
LT*β*R^−/−^ animals show more and larger inflammatory areas in the (a) lung and (b) liver 7 and 21 days after infection with *T. gondii* (ME49) cysts compared to WT animals. The lung and liver were isolated from uninfected control mice 7 and 21 days after i.p. infection with 40 *T. gondii* (ME49) cysts and fixed in formalin. Tissues were embedded in paraffin, 10 *μ*m sections were generated, and HE staining was performed. Original magnification as indicated. 3 animals were analyzed for each time point, and a representative section from one organ is shown in each case. Arrows indicate small, dense lymphocyte infiltrates that are considered part of the basal LT*β*R^−/−^ phenotype. Arrowheads indicate inflammatory infiltrates seen in infected animals.

To analyze tissue pathology, formalin-fixed, paraffin-embedded, and HE-stained tissue sections (10 *μ*m) from the lung and liver were assayed for inflammatory infiltrates (Figure 2). It is important to note that in uninfected/untreated LT*β*R^−/−^ animals, lymphocyte infiltrates have been described in the kidneys, lungs, liver, pancreas, submandibular glands, mesenterium, cortex of the suprarenal glands, and fatty tissue of the mediastinum [40] and could accordingly be observed in the lungs (Figure 2(a)) of uninfected LT*β*R^−/−^animals. In addition to these small infiltrates, LT*β*R^−/−^ lungs showed large inflammatory infiltrates on days 7 and 21 after infection. In contrast, only very few such inflammatory infiltrates could be found in the lungs of WT littermates on days 7 and 21 and they tended to be considerably smaller and less dense (Figure 2(a)). Similarly, the livers of uninfected LT*β*R^−/−^ mice were characterized by small lymphocyte infiltrates which could not be found in WT livers (Figure 2(b)). On day 7 p.i., the LT*β*R^−/−^ livers show a marked increase of infiltrates, whereas in the livers of WT mice, the number of inflammatory infiltrates is much lower. By day 21, the LT*β*R^−/−^ livers still showed considerable number of inflammatory infiltrates, while these have disappeared from the livers of WT mice. These findings are quantified and summarized in Table 2, showing that in the lungs of WT animals, inflammatory infiltrates could mainly be observed on days 7 and 12. In contrast, these infiltrates are much more persistent in LT*β*R^−/−^ mice: they were observed from day 3 through day 36 in the lungs. Findings were similar in the livers: infiltrates were detected in WT animals mainly on days 7 and 12, while they could be observed in LT*β*R^−/−^ animals from day 5 through day 14. Thus, organ pathology was much more pronounced and persisted for a longer period of time in LT*β*R^−/−^ compared to WT animals.

### 3.3. LT*β*R^−/−^ Animals Have Higher and More Persistent Cyst Count

**Figure 3:**
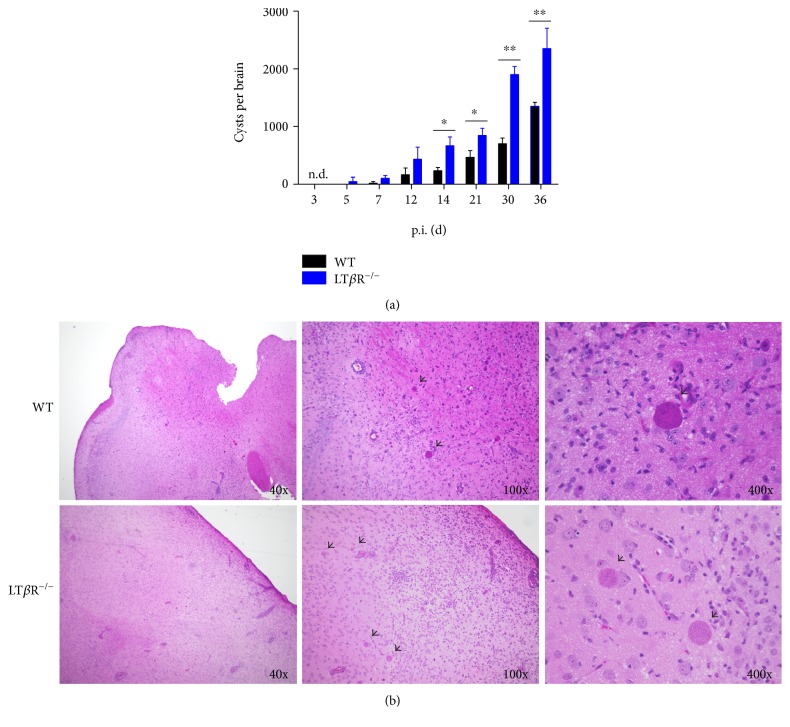
Analysis of parasite burden in the brain of WT and LT*β*R^−/−^ animals. Animals were infected i.p. with 40 cysts of *T. gondii* (ME49), sacrificed on the days indicated, and the brains were prepared. One hemisphere was used for isolation of cysts, which were isolated by mincing the tissue with a scalpel and then passing it through consecutively higher gauge cannulas, followed by two centrifugation steps to first remove pelleted cells and tissue debris and then pellet the cysts. One half of the second hemisphere was used to generate HE stains from paraffin sections after formalin fixing of tissue. (a) Cysts per brain were calculated by multiplying cyst number counted in one hemisphere by two (*n* = 3 in all cases, except day 30 and day 36 from LT*β*R^−/−^ animals, where only 2 animals were analyzed). (b) Cysts (arrows) in HE-stained brain sections 60 days after i.p. infection with *T. gondii* (ME49) are shown. One representative section of brain tissue from one of three animals is shown. Original magnifications as indicated. ^∗^*p* < 0.05, ^∗∗^*p* < 0.01.

To determine whether LT*β*R^−/−^ mice showed differences in the progression into and through the chronic phase of *T. gondii* infection, bradyzoite containing cysts were counted in HE sections of liver, lung, and brain (Table 3). Cysts first appeared in the lungs of LT*β*R^−/−^ mice on day 5 and could be observed on days 7, 12, and 14. In contrast, in the lungs of WT mice cysts could only be found on day 14. While cysts appeared in the liver in both genotypes on day 7 and persisted only slightly longer in LT*β*R^−/−^ animals compared to WT animals (days 14 and 12, respectively), the number of cysts was elevated in the LT*β*R^−/−^ mice. Differences in cyst counts were most obvious in the brain. While cysts appeared at the same time after infection (day 14), actual numbers were much higher in LT*β*R^−/−^ animals than in WT animals (13–18 versus 2–5, respectively, on day 36). The increased presence of cysts in the brain of LT*β*R^−/−^ mice was confirmed by isolating and counting cysts from the brains (Figure 3(a)). Formalin-fixed, paraffin-embedded, and HE-stained tissue sections also showed an increased presence of cysts in brains of LT*β*R^−/−^ mice (Figure 3(b)). While disease progression (entry into the acute phase and progression into the chronic phase) apparently occurred within a similar time frame in both genotypes, LT*β*R^−/−^ animals were less able to contain reproduction of the parasites, leading to a more pronounced tissue pathology, higher cyst numbers, and longer persistence of cysts.

### 3.4. LT*β*R^−/−^ Mice Do Not Show Splenic Enlargement and Increase in Splenic Cell Count after Infection with *T. gondii*

**Figure 4:**
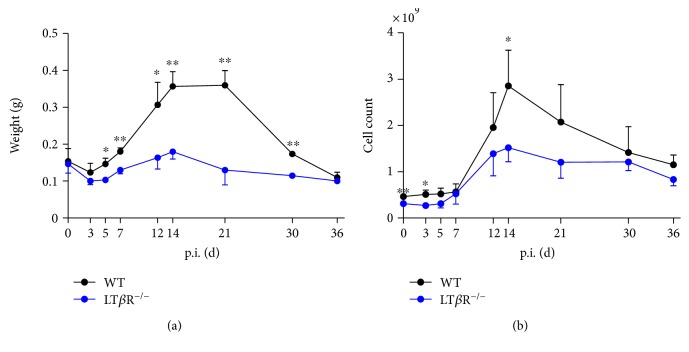
Splenomegaly is observed only in WT but not in LT*β*R^−/−^ animals after infection with *T. gondii* (ME49). Mice were infected with 40 cysts and sacrificed on the days indicated. Controls were uninfected animals. (a) Spleens were isolated and weighed. (b) Cell numbers were determined by mincing and homogenizing the spleen, passing the obtained cell supension through a 40 *μ*m cell strainer and counting live cells (*n* = 3 in all cases except day 30 and day 36 from LT*β*R^−/−^ animals, where only 2 animals were analyzed). ^∗^*p* < 0.05, ^∗∗^*p* < 0.01.

To assess the inflammatory response in LT*β*R^−/−^ mice after *T. gondii* infection, spleen weight was analyzed. In WT mice, a roughly twofold increase of spleen weight during acute infection could be found which returned to preinfection levels by day 36. In contrast, in LT*β*R^−/−^ mice, spleen weight increased only marginally during acute infection and returned to physiological levels by day 21 (Figure 4(a)). Splenic cell counts peaked on day 14 both in WT and LT*β*R^−/−^ animals, but were significantly lower in the latter (Figure 4(b)).

### 3.5. LT*β*R^−/−^ Mice Show Minor Alterations in Various Tissue Injury Parameters

**Figure 5:**
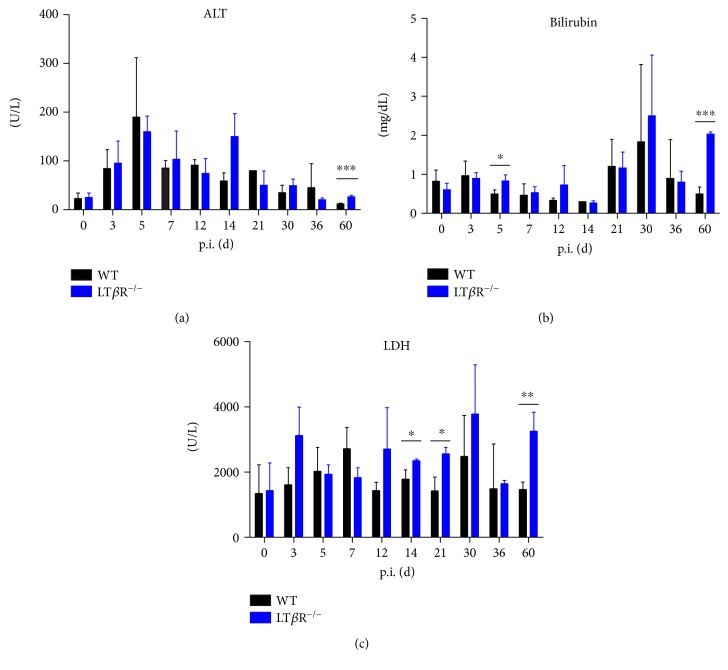
Serum parameters in WT and LT*β*R^−/−^ animals. Mice were infected with 40 cysts of *T. gondii* (ME49) and sacrificed on the days indicated. Controls were uninfected animals. Serum was obtained by accessing the vena cava inferior, bleeding the animals, and removing cells by centrifugation after allowing a suitable time for clotting. Analysis was performed on a Spotchem 4430. (a) ALT, (b) bilirubin, and (c) LDH (*n* = 3 in all cases except day 30 and day 36 from LT*β*R^−/−^ animals, where only 2 animals were analyzed). ^∗^*p* < 0.05, ^∗∗^*p* < 0.01, and ^∗∗∗^*p* < 0.001.

Alanine transaminase (ALT) levels were measured to determine liver stress after *T. gondii* infection (Figure 5(a)). In WT animals, ALT levels rose quickly until day 7 p.i., then gradually dropped to preinfection levels by day 60 p.i. ALT levels of LT*β*R^−/−^ animals progressed in a similar manner, except for a marked but not significant transient increase on day 14. On day 60, ALT levels were significantly higher in LT*β*R^−/−^ compared to WT animals. Bilirubin is also considered to indicate liver damage. Interestingly, after infection with *T. gondii*, bilirubin levels did not markedly change early during infection (Figure 5(b)), although levels were slightly but significantly increased in LT*β*R^−/−^ animals on day 5 p.i. Later in infection (days 21 and 30), an increase in bilirubin levels could be observed in both genotypes. On day 60, LT*β*R^−/−^ animals again show a significant increase in bilirubin compared to WT animals. Since increased LDH is an indicator of cell destruction, LDH levels were determined. Only a slight increase in LDH levels was measured in WT animals throughout the course of infection, with the exceptions of day 7 and day 30 p.i., when a moderate increase occurred. LDH levels of LT*β*R^−/−^ animals tended to be higher, with a significant increase on days 14, 21, and 60 (Figure 5(c)).

### 3.6. LT*β*R^−/−^ Mice Show Lacking or Delayed Cytokine Responses after Infection with T. gondii

**Figure 6:**
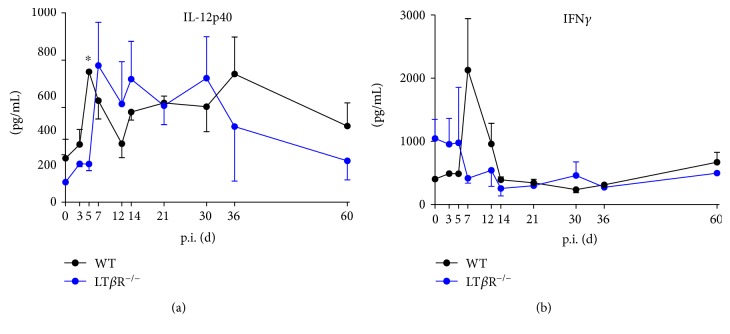
Cytokine production is disturbed in LT*β*R^−/−^ animals. 50 *μ*L of murine serum was collected from uninfected and infected WT and LT*β*R^−/−^ animals (*T. gondii* (ME49), 40 cysts) on the days indicated. (a) IL-12p4 and (b) IFN*γ* amounts were determined by ELISA. (*n* = 3 in all cases except day 30 and day 36 from LT*β*R^−/−^ animals, where only 2 animals were analyzed). ^∗^*p* < 0.05.

**Figure 7:**
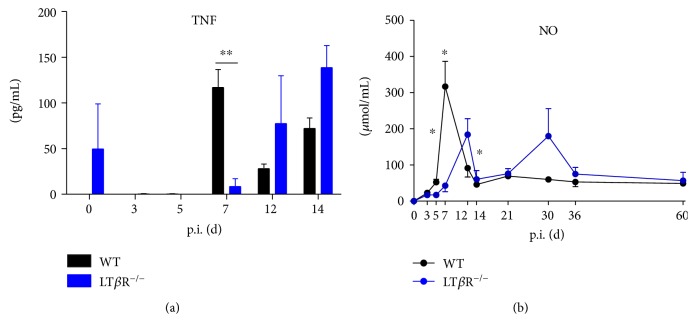
Compared to WT animals, LT*β*R^−/−^ animals show delayed increase of TNF*α* in the serum in the acute phase of infection with *T. gondii*. 50 *μ*L of murine serum was collected from uninfected and infected WT and LT*β*R^−/−^ animals, TNF*α* levels were determined by ELISA (a), and nitric oxide levels were determined by colorimetric detection of nitrite after conversion of nitrate to nitrite (b). (*n* = 3 in all cases except d 0 (both genotypes) and d 14 (LT*β*R^−/−^), where only 2 animals were analyzed). ^∗^*p* < 0.05, ^∗∗^*p* < 0.01.

Secretion of IL-12 by macrophages and DC is one of the initial steps in the innate immune response to *T. gondii* and induces release of IFN*γ* by NK and T cells [7]. Compared to LT*β*R^−/−^ animals, WT animals were observed to have significantly increased levels of serum IL-12p40 by day 5, whereas LT*β*R^−/−^ animals exhibited this increase two days later (Figure 6(a)). Interestingly, although slightly higher amounts of IFN*γ* could be found in LT*β*R^−/−^ compared to WT animals before infection, these amounts did not increase after infection, as seen in WT animals, where levels rose about 4-fold (Figure 6(b)). Despite this marked increase, the difference was not significant, probably due to the high variance found in LT*β*R^−/−^ animals. TNF is another cytokine that is secreted by macrophages early in infection [7]. While WT animals showed a marked increase of TNF already on day 7 p.i., LT*β*R^−/−^ animals initially exhibited significantly lower TNF levels which reached WT levels only on day 14 p.i. (Figure 7(a)). As NO produced by macophages is considered to be an important microbicidal mechanism in the innate immune response to *T. gondii* [42], total NO in serum of WT and LT*β*R^−/−^ mice was analyzed.  Figure 7(b) reveals a strong and transient increase of serum NO in WT on day 7 p.i. For the remainder of the observation period, serum NO levels remain moderately elevated in WT animals. In contrast, LT*β*R^−/−^ animals showed a delayed and reduced increase of serum NO levels on day 12 p.i. and an additional similar peak on day 30 p.i. that could not be detected in WT animals.

### 3.7. Differential Expression of Genes Involved in Early Innate Immune Response to *T. gondii*

**Figure 8:**
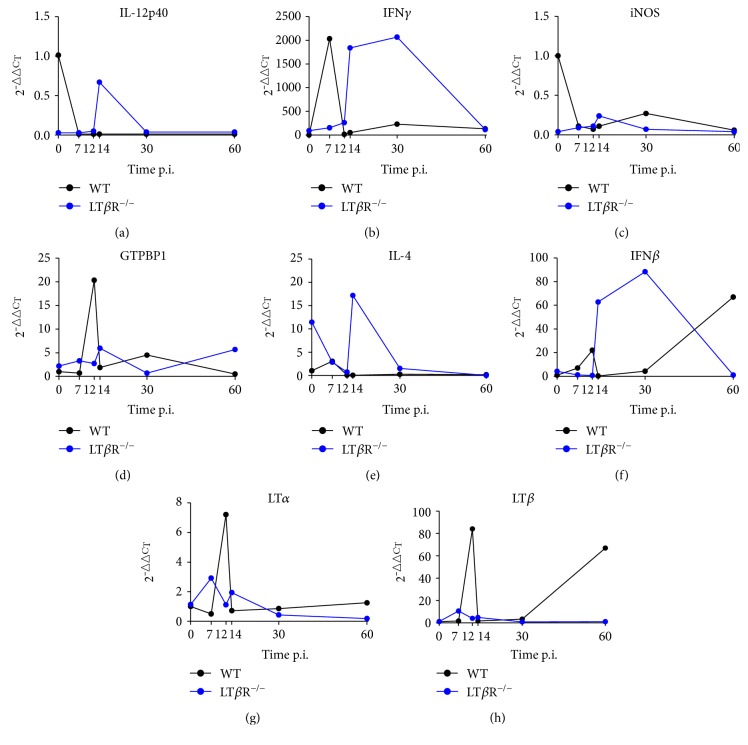
LT*β*R^−/−^ animals show differential expression of immune relevant genes in the lung in comparison to WT animals after infection with *T. gondii* (ME49). Mice were sacrificed, RNA was isolated from the lungs from uninfected and infected WT and LT*β*R^−/−^ animals on the days indicated, and expression levels were determined via quantitative RT-PCR. (a) IL-12p40, (b) IFN*γ*, (c) iNOS, (d) GTPBP1, (e) IL-4, (f) IFN*β*, (g) LT*α*, and (h) LT*β*. (*n* = 3 in all cases except day 30 and day 36 from LT*β*R^−/−^ animals, where only 2 animals were analyzed).

Expression levels of IL-12p40, IFN*γ*, GTP-binding protein 1 (GTPBP1), IL-4, IFN*β*, LT*α*, and LT*β* in the lungs of WT and LT*β*R^−/−^ animals after *T. gondii* infection were compared. Expression levels for IL-12p40 decreased in WT animals by day 7 p.i, whereas LT*β*R^−/−^ animals showed much lower expression levels compared to WT animals before infection, but a transient increase in IL-12p40 expression on day 14 p.i. (Figure 8(a)). 7 days after infection with *T. gondii*, IFN*γ* expression levels increased dramatically in WT animals, returned to normal by day 12, and showed only a mild increase during the further course of infection (Figure 8(b)). In contrast, in LT*β*R^−/−^ animals, IFN*γ* levels did not increase until day 14, but then reached levels comparable to WT animals. Also, IFN*γ* levels remained high at least up to day 40 p.i. and only returned to slightly higher than normal levels by day 60. On the other hand, expression of induced nitric oxide synthase (iNOS) was much lower in LT*β*R^−/−^ animals compared to WT animals before infection and did not increase markedly after infection (Figure 8(c)). In WT animals, iNOS expression decreased after infection and remained at low levels at least until day 60 p.i. Expression of GTPBP1 increased transiently but markedly in WT animals on day 12 p.i and then remained at slightly elevated levels (Figure 8(d)). LT*β*R^−/−^ animals did not exhibit such a distinct increase p.i.; GTPBP1 expression levels were only moderately increased during the course of infection. WT animals showed only a slight (around 2-fold) and transient increase of IL-4 expression 7 days p.i. (Figure 8(e)). Of note, IL-4 expression in LT*β*R^−/−^ animals was increased more than 10-fold before infection when compared to WT animals and this expression decreased markedly early after infection (days 7 and 12), followed by a distinct but transient increase on day 14 p.i. IFN*β* expression levels in WT animals showed a 20-fold increase on day 12 p.i. (Figure 8(f)). Then levels dropped again, but rose about 70-fold between days 30 and 60 levels. In LT*β*R^−/−^ animals, INF*β* levels remained low until day 12, but steeply increased on day 14 (60-fold), remained at this level until day 30, but then dropped to normal titers again by day 60. Expression patterns of LT*α* and LT*β* were similar (Figures 8(g) and 8(h)): expression in WT animals exhibited a distinct peak on day 12 (approximately 8-fold for LT*α* and approximately 80-fold for LT*β*), whereas expression in LT*β*R^−/−^ animals was only moderately increased.

### 3.8. IFN*γ*-Induced Expression of mGBPs Is Strikingly Reduced in LT*β*R^−/−^ Animals

**Figure 9:**
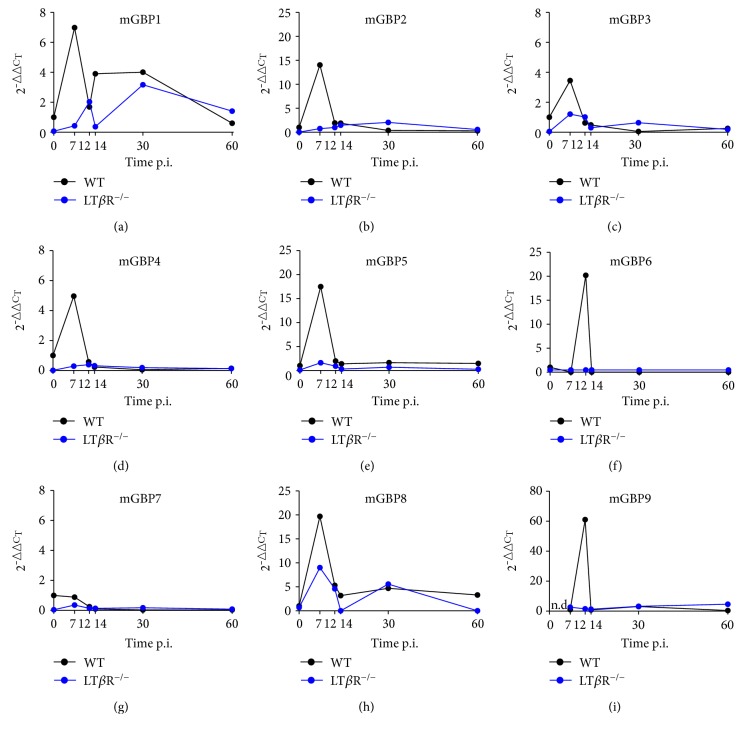
LT*β*R^−/−^ animals show abrogated or delayed expression of mGBP genes in comparison to WT animals after infection with *T. gondii* (ME49). Mice were sacrificed, RNA was isolated from lungs from uninfected and infected WT and LT*β*R^−/−^ animals on the days indicated, and expression levels were determined via quantitative RT-PCR. (a) mGBP1, (b) mGBP2, (c) mGBP3, (d) mGBP4, (e) mGBP5, (f) mGBP6, (g) mGBP7, (h) mGBP8, and (i) mGBP9 (*n* = 3 in all cases except day 30 and day 36 from LT*β*R^−/−^ animals, where only 2 animals were analyzed).

mGBPs play an important role in the immune defense against *T. gondii* and are prominent IFN*γ*-induced genes [35]. Analysis of mGBP expression in the lung after *T. gondii* infection revealed a consistent picture (Figure 9). Generally, mGBP expression before infection tended to be lower in LT*β*R^−/−^ animals. Early after infection, expression of most mGBPs was increased transiently, but markedly in WT animals. Exceptions were mGBP1 (Figure 9(a)), where a second increase of expression could be observed later in infection and mGBP7 (Figure 9(g)) where no increase of expression levels could be observed. In contrast, the expression of mGBPs in LT*β*R^−/−^ animals either remained more or less at levels before infection (mGBP2, mGBP4, mGBP5, mGBP6, and mGBP9) or the increase was much lower (mGBP3 and mGBP8) or lower and delayed (mGBP1) when compared to WT animals. Similar to WT animals, no expression of mGBP7 could be observed in LT*β*R^−/−^ animals. Analysis of spleen tissue showed a similar absence of mGBP expression in LT*β*R^−/−^ animals compared to WT animals after *T. gondii* infection (data not shown). Taken together, these results strongly suggest that LT*β*R-initiated upregulation of immune relevant genes, most notably mGBPs, is essential for the survival of *T. gondii* infection.

## 4. Discussion

To date, there has been no evidence for a role of the LT*β*R in the immune defense to *T. gondii*. The present study clearly demonstrates substantially reduced overall survival of *T. gondii* infection in LT*β*R^−/−^ mice which begins to succumb to the infection around day 12. Around 50% of the LT*β*R-deficient animals survive the acute phase of the *T. gondii* infection and are able to progress into the chronic phase of the disease before survival rates drop again. LT*β*R^−/−^ mice fail to induce IFN*γ*, and mGBPs are subsequently not upregulated, leading to a breakdown of the antitoxoplasma immune response. These results point towards a major role for the LT*β*R in an efficient immune response to *T. gondii* and are in accordance with other studies suggesting that the LT*β*R acts as an important immune regulator, not only in bacterial infection models for listeriosis or tuberculosis [5, 43, 44] but also in intracellular parasite infection models for malaria [45, 46] or leishmaniasis [47–50]. The role of the LT*β*R in these disease models is quite diverse. In infection models with *L. monocytogenes* and *M. tuberculosis*, LT*β*R^−/−^ mice not only show a delayed/abrogated activation of the innate immune response [5, 44] but also an absence of specific T cell responses [43]. In cutaneous leishmaniasis, the presence of peripheral lymph nodes (LN) is essential for driving a T_H_1 response and the absence of all LN in LT*β*R^−/−^ mice leads to a marked susceptibility to the disease [48], whereas in visceral leishmaniasis, signaling through the LT*β*R is protective via promoting DC development and maturation [47]. The current model is that the immune response to *T. gondii* is initiated by activation of DCs via TLR11/12 MyD88 interaction after recognition of the protozoan profilin-like protein [51]. Downstream signaling via the canonical NF*κ*B pathway then leads to secretion of IL-12 by DCs which in turn induces NK cells to release IFN*γ*. Since LT*β*R signaling occurs via the classic and the alternative NF*κ*B signaling pathway, it might be envisaged that LT*β*R^−/−^ animals show delay in IL-12p40 secretion. Interestingly, Xu et al. [49] have demonstrated that blocking of LT*β*R signaling via HVEM-Ig or LT*β*R-Ig leads to defective IL12p40 production and increased susceptibility to *Leishmania major infection.* It can be speculated therefore that cooperation of LT*β*R and TNFRp55 signaling pathways is required for an efficient immune response to *T. gondii*. Since LT*α*_1_*β*_2_^−/−^ mice do not succumb to *L. major* infection, LIGHT seems to be the relevant LT*β*R ligand in this case. Therefore, the susceptibility of TNFRp55^−/−^, LIGHT^−/−^, and functional LT*β*R/TNFRp55 doubly deficient mice to *T. gondii* is being studied to evaluate to what extent either pathway and which ligands are required for an efficient immune response. Furthermore, imperfect DC differentiation might be responsible for a diminished IL-12 production (see below) in LT*β*R^−/−^ mice [52]. Interestingly, expression of the LT*β*R is essential for the development of experimental cerebral malaria (ECM) after infection with *Plasmodium berghei* ANKA and prolongs survival in LT*β*R^−/−^-deficient mice due to their inability to generate an effective (CD8^+^) T cell response, which is responsible for ECM pathophysiology [53, 54]. These findings are explained by the role that LT*β*R signaling plays in the development and homeostasis of the secondary lymphoid organs [40], its essential role in optimizing DC maturation and function, in supporting CD4 T cell maturation, and its ability to polarize T cells [52, 55]. IFN type I and type II have been shown to be important for survival of viral and nonviral infections [31, 56]. In the defense against MCMV, LT*β*R signaling has been demonstrated to initiate the type I IFN response [57, 58]. In listeria and mycobacteria infections, LT*β*R signaling has been shown to induce IFN type I and type II responses [5, 22, 44, 49, 59]. In toxoplasmosis, recognition of parasitic profilin via toll like receptors 11 and 12 is one of the major signals triggering IL-12 production in DC which in turn induces IFN*γ* production by NK cells [22, 60–62]. Here, in *T. gondii* infected LT*β*R^−/−^ mice, a delayed increase of serum IL-12p40 and a failure to upregulate serum IFN*γ* levels could be demonstrated. IFN*γ* signaling is essential for an efficient antitoxoplasma immune response since neither IFN*γ*^−/−^ nor IFN*γ*R^−/−^ mice are able to efficiently contain *T. gondii* infections and die early during the acute phase [62, 63]. IFN*γ* triggers several antiparasitic mechanisms including the induction of iNOS which leads to elevated levels of microbicidal NO and the induction of mGBP expression, both of which play an important role in the host defense against *T. gondii* [22, 35, 36, 64, 65]. LT*β*R^−/−^ mice show a delayed increase of serum NO levels. Compared to WT mice, induction of mGBPs was virtually absent. Recently, members of the mGBP family have been shown to be important for survival after *T. gondii* infection [35–37, 39]. Interestingly, mGBPs are IFN*γ* and, to a lesser degree, IFN type I responsive genes [35]. Most mGBP proteins are rapidly recruited to the *T. gondii* parasitophorous vacuole in *T. gondii*-infected cells, and expression of at least mGBP2 is required for efficient elimination of the parasite [36, 39]. The marked failure of mGBP family member induction in LT*β*R^−/−^ mice therefore provides an explanation for the high mortality observed. In addition, WT mice exhibit splenomegaly due to increased cell numbers in the spleen. In contrast, spleen weights and cell numbers increase to a significantly lesser degree in LT*β*R^−/−^ mice. It has been described previously that LT*α*/*β*-LT*β*R signaling is activated in *T. gondii*-infected WT mice and may, at least in part, be responsible for modulating spleen architecture and organization via chemokine modulation [66]. It has been shown that in LT*β*R^−/−^ mice, peripheral lymphoid organs, Peyer's patches, and gut-associated lymphoid tissue are absent [40]. Furthermore, dendritic cell (DC) maturation is impaired in these animals [52, 67, 68]. To address the question whether the susceptibility of LT*β*R^−/−^ mice to *T. gondii* infection is due to the lack of adequate priming of immature T cells by DC, further studies are required, for example, using bone marrow chimera models [69]. In addition, since LT*β*R^−/−^ animals also lack B cell follicles in the spleen [40, 70], it will be interesting to see whether these mice are able to mount a *T. gondii*-specific antibody response and develop an antigen-specific T cell response. The failure to mount an effective specific T and B cell response against *T. gondii* and the possible inability to drive the parasite into its chronic stage and/or to prevent reactivation of chronic toxoplasmosis might explain the higher parasite numbers observed in the brains of LT*β*R^−/−^ animals and concurs with the increased parasitemia described in LT*β*R^−/−^ animals in the ECM model by other groups [53, 54]. Taken together, this underscores the importance of LT*β*R signaling in innate as well as adaptive immunity. We therefore speculate that LT*β*R signaling is necessary for either driving *T. gondii* infection into the chronic stage or maintaining this chronic stage, and further analysis of the role of the LT*β*R in this context may lead to a better understanding of the mechanisms of *T. gondii* stage conversion.

## 5. Conclusions

These data demonstrate that beyond being responsible for the development of secondary lymphatic organs, which provide the environment required to mount an efficient adaptive immune response, LT*β*R signaling modulates these responses which are important for establishing and maintaining chronic toxoplasmosis and the LT*β*R is necessary, via inducing an IFN type II response, for initiating innate effector mechanisms essential for containing acute *T. gondii* infection.

## Figures and Tables

**Table 1:** Primer and probe sequences for RT-PCR.

Target	Primers	Probe
*β*-Actin	5′TGACAGGATGCAGAAGGAGA 3′CGCTCAGGAGGAGCAATG	^a^106
mGBP1	5′CAGACTCCTGGAAAGGGACTC 3′CTTGGACCTGGAACATTCACTGAC	^a^41
mGBP2	5′TGAGTACCTGGAACATTCACTGAC 3′AGTCGCGGCTCATTAAAGC	^a^17
mGBP3	5′GGCTGAGGACTGTCCCTGT 3′CATGGTCCACTCGGAAGC	^a^21
mBGP4	5′GCCAAGATCAAGACCCTCAG 3′CCACGTAGGTTGTCACCAGA	^a^48
mGBP5	5′TCACTGAAGCTGAAGCAAGG 3′GCGTCAAAAACAAAGCATTTC	^a^48
mGBP6	5′ATATTTCAACATTTTTTGTTCCTTGT 3′GAAATGGGAGAAAAAATAAATGAAGC	FAM-AGTCATGTTCAATCTTCTCCCTCTTGTCC-DB
mGBP7	5′GCAGAGAATCCGGTGCAG 3′TTTCCACTAGGCACACAGGA	^a^93
mGBP8	5′AAGAAGCTGAAGGAACAAAAGGC 3′GAAATGGGAGAAAAAATAAATGAAGC	FAM-TGTTTCAGTTGCTGTATCTCTCCGTCCA-TMR
mGBP9	5′TTCCAAAACTTTCTCCAGTCACAGTA 3′GGCACGCTCCTCTGCAA	FAM-CCAGCAGTGAGGGCTCTATCTGCCT-TMR
GTPBP1	5′GGTGCAGAGCAAAGATGATG 3′ATCTGGAATATCGGGCACAT	^a^75
IL-4	5′CATCGGCATTTTGAACGAG 3′CGAGCTCACTCTCTGTGGTG	^a^2
IL-12p40	5′GATTCAGACTCCAGGGGACA 3′TGGTTAGCTTCTGAGGACACATC	^a^27
iNOS	5′CTTTGCCACGGACGAGAC 3′TCATTGTACTCTGAGGGCTGAC	^a^13
LT*α*	5′TCCCTCAGAAGCACTTGACC 3′GAGTTCTGCTTGCTGGGGTA	^a^62
LT*β*	5′CCTGGTGACCCTGTTGTTG 3′TGCTCCTGAGCCAATGATCT	^a^76
IFN*β*	5′CAGGCAACCTTTAAGCATCAG 3′CCTTTGACCTTTCAAATGCAG	^a^95

^a^Numbers identify probes obtained from the Roche Universal ProbeLibrary (Roche).

**Table 2:** Inflammatory infiltrates in the lung and liver.

Organ	Genotype	Days p.i.								
		0	3	5	7	12	14	21	30	36
Lung	WT	−	−	−	+	++	−	(+)	−	−
	^a^LT*β*R^−/−^	−	+++	+++	+++	+++	+++	+++	+++	++
Liver	WT	−	−	−	++	++	−	+	−	−
	^a^LT*β*R^−/−^	−	−	++	++	+++	+	−	−	−

The number of inflammatory infiltrates per visual field were scored in HE-stained sections, at least 10 visual fields were evaluated per slide. No infiltrates: −; 1–3 infiltrates: (+); 4–8 infiltrates: +; 9–12 infiltrates: ++; 13–18 infiltrates: +++. ^a^Infiltrates considered to be part of the basal LT*β*R^−/−^ phenotype were not included in the scoring.

**Table 3:** Cyst count in the liver and lung.

Organ	Genotype	Days p.i.								
		0	3	5	7	12	14	21	30	36
Lung	WT	—	—	—	—	—	2	—	—	—
	LT*β*R^−/−^	—	—	0.5	1	2	1	—	—	—
Liver	WT	—	—	—	3	2.5	—	—	—	—
	LT*β*R^−/−^	—	—	—	2.5	2	6	—	—	—
Brain	WT	—	—	—	—	—	0.5	1	1.5	3
	LT*β*R^−/−^	—	—	—	—	—	2.5	2.5	10.5	16.5

Organ sections from 3 animals per time point were evaluated, except on day 30 and day 36 from LT*β*R^−/−^ animals, where only 2 animals were evaluated. The number of cysts per organ section was counted.

## Abbreviations

AST: Aspartate transaminase
DC: Dendritic cell
ECM: Experimental cerebral malaria
GBP: Guanylate-binding protein
GTPBP1: GTP-binding protein 1
iNOS: Induced nitric oxide synthase
IRG: Immunity-related gene 1
LDH: Lactate dehydrogenase
LN: Lymph node
LT: Lymphotoxin
LT*β*R: Lymphotoxin beta receptor
mGBP: Murine guanylate-binding protein
p.i.: Post infection
TNFR: TNF receptor
WT: Wild type.
